# In-silico analysis of potent Mosquirix vaccine adjuvant leads

**DOI:** 10.1186/s43141-023-00590-x

**Published:** 2023-11-30

**Authors:** Okello Harrison Onyango, Cynthia Mugo Mwenda, Grace Gitau, John Muoma, Patrick Okoth

**Affiliations:** 1 Department of Biological Sciences (Molecular Biology, Computational Biology, and Bioinformatics Section), School of Natural and Applied Sciences, Masinde Muliro University of Science and Technology, P.O. BOX 190-50100, Kakamega, Kenya; 2 Department of Biological Sciences, School of Pure and Applied Sciences, Meru University of Science and Technology, P.O. BOX 972-60200, Meru, Kenya; 3 Department of Biochemistry and Biotechnology, School of Biological and Life Sciences, The Technical University of Kenya, P.O. BOX 52428-00200, Nairobi, Kenya

**Keywords:** Mosquirix vaccine, In-silico, Natural compounds, Vaccine adjuvant, Malaria, Hierarchical virtual screening, Molecular dynamics simulation, Molecular docking

## Abstract

**Background:**

World Health Organization recommend the use of malaria vaccine, Mosquirix, as a malaria prevention strategy. However, Mosquirix has failed to reduce the global burden of malaria because of its inefficacy. The Mosquirix vaccine’s modest effectiveness against malaria, 36% among kids aged 5 to 17 months who need at least four doses, fails to aid malaria eradication. Therefore, highly effective and efficacious malaria vaccines are required. The well-characterized *P*. *falciparum* circumsporozoite surface protein can be used to discover adjuvants that can increase the efficacy of Mosquirix. Therefore, the study sought to undertake an in-silico discovery of *Plasmodium falciparum* circumsporozoite surface protein inhibitors with pharmacological properties on Mosquirix using hierarchical virtual screening and molecular dynamics simulation.

**Results:**

Monoclonal antibody L9, an anti-*Plasmodium falciparum* circumsporozoite surface protein molecule, was used to identify *Plasmodium falciparum* circumsporozoite surface protein inhibitors with pharmacological properties on Mosquirix during a virtual screening process in ZINCPHARMER that yielded 23 hits. After drug-likeness and absorption, distribution, metabolism, excretion, and toxicity property analysis in the SwissADME web server, only 9 of the 23 hits satisfied the requirements. The 9 compounds were docked with *Plasmodium falciparum* circumsporozoite surface protein using the PyRx software to understand their interactions. ZINC25374360 (−8.1 kcal/mol), ZINC40144754 (−8.3 kcal/mol), and ZINC71996727 (−8.9 kcal/mol) bound strongly to *Plasmodium falciparum* circumsporozoite surface protein with binding affinities of less than −8.0 kcal/mol. The stability of these molecularly docked *Plasmodium falciparum* circumsporozoite surface protein-inhibitor complexes were assessed through molecular dynamics simulation using GROMACS 2022. ZINC25374360 and ZINC71996727 formed stable complexes with *Plasmodium falciparum* circumsporozoite surface protein. They were subjected to in vitro validation for their inhibitory potential. The IC_50_ values ranging between 250 and 350 ng/ml suggest inhibition of parasite development.

**Conclusion:**

Therefore, the two *Plasmodium falciparum* circumsporozoite surface protein inhibitors can be used as vaccine adjuvants to increase the efficacy of the existing Mosquirix vaccine. Nevertheless, additional *in vivo* tests, structural optimization studies, and homogenization analysis are essential to determine the anti-plasmodial action of these adjuvants in humans.

## Background

Malaria is a global health burden affecting millions of people annually. In 2020, World Health Organization (WHO) reported roughly 241 million cases and 627,000 deaths, an increase of almost 14 million cases and 69,000 deaths from the 2019 malaria statistics [52]. Stofberg et al. [41] projected 229 million infections and more than 419,000 fatalities worldwide in 2019. The figures for malaria deaths in 2019 surpassed this projection, indicating that the disease persists as a significant public health burden. Africa contributed 95% of malaria cases and 96% of deaths to the global malaria figures in 2020 [52]; 95% of malaria-related deaths occur in sub-Saharan Africa, and 67% involve kids aged 5 years and below [41]. Malaria is caused by the *Plasmodium* species, a single-celled eukaryote protozoan parasite in the phylum Apicomplexa. The most severe manifestation of the illness in humans is caused by *P. falciparum* [2]. Due to the parasite’s complicated life cycle and lack of effective malaria vaccines and medicines, efforts to eradicate malaria have been limited and curtailed.

**Fig. 1:**
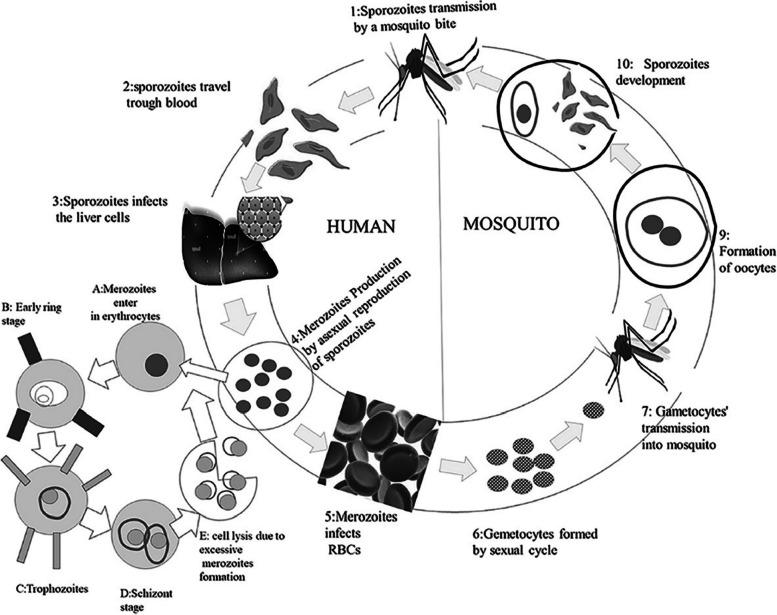
*P. falciparum* life cycle. The cycle shows how malaria is transmitted from infected mosquitoes to humans [30]

Within the vertebrate human host and the vector, the female *Anopheles* mosquito, *Plasmodium falciparum,* leads a highly complex life cycle, Fig. 1 [30]. After a blood meal, an infected mosquito injects the sporozoites into the dermis, where they travel to the bloodstream and cause the unicellular protozoan parasites to infect people. The sporozoites travel through the blood and enter hepatocytes, where they begin to reproduce asexually for the first time [24]. Merozoites are finally delivered into the bloodstream when the infested hepatocytes burst. They infect RBCs to start the intra-erythrocytic growth phase. The *P*. *falciparum* first develops into the ring phase, then into trophozoites, and finally, schizonts, which burst to release merozoites that reinvade fresh RBCs. A tiny number of the ring-stage parasites in the infected RBC (iRBC) may also mature sexually to become gametocytes. By ingesting them during a blood meal, mosquitoes continue the cycle of transmission by producing gametes that initiate the cycle of sporogony inside the mosquito [41].

The erythrocytic cycle of the parasite’s life cycle is mainly linked to malaria symptoms; for instance, in *P. falciparum*, the adhesion phenomenon is connected to the severity of the illness [26]. This is explained by the fact that the parasite and iRBC intracellular material are released when schizonts burst. The intracellular substances and exposed parasites trigger immunological reactions that aid in the pathogenesis of malaria [41]. The change from the 25°C poikilothermic vectors to the 37 °C homoeothermic host places the parasites in a harsh environment that they must withstand to complete their intricate life cycle. Thermal stress is created as a result and exacerbated during fever episodes marked by temperature increases up to 41 °C [47].

**Fig. 2:**
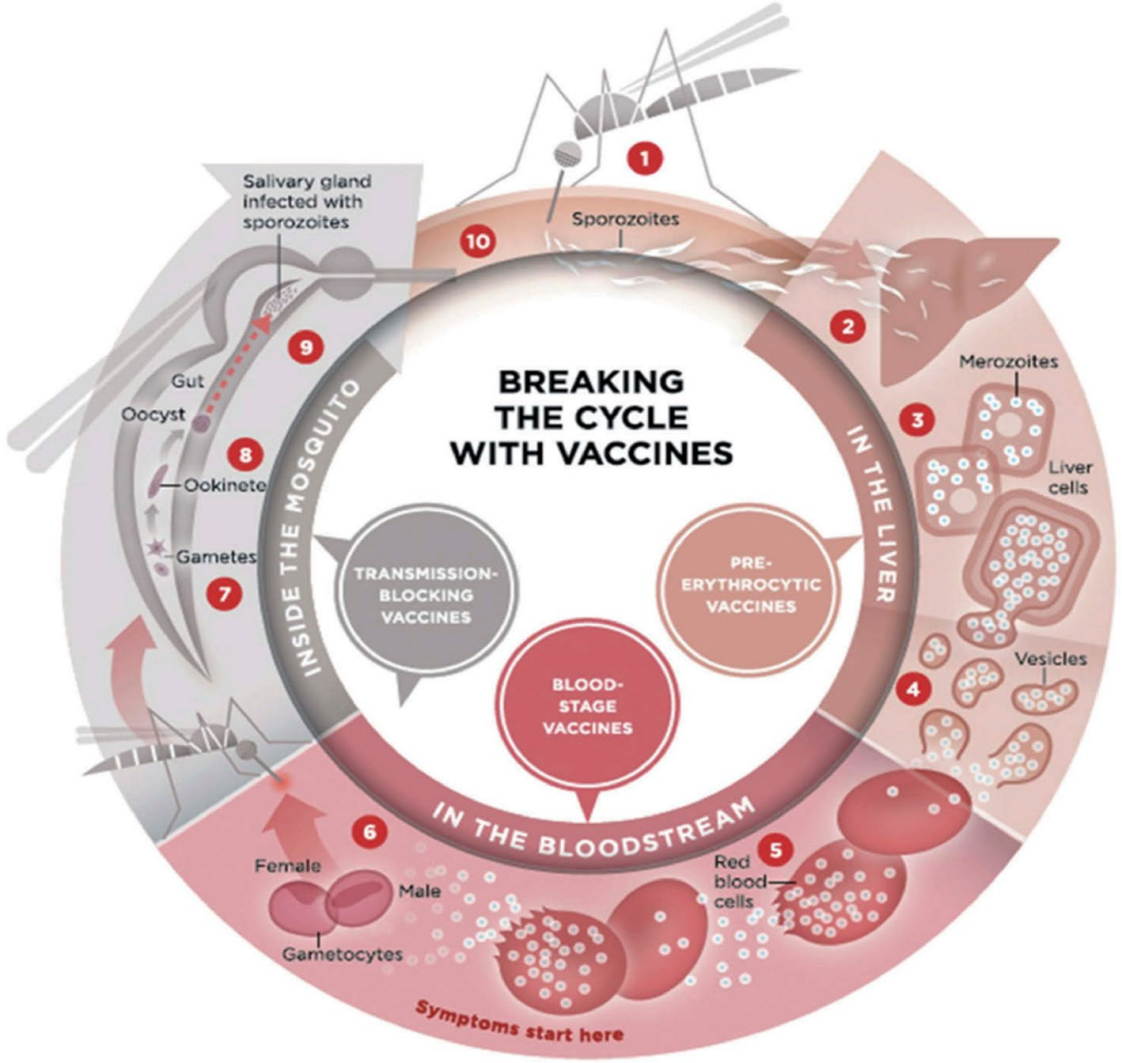
Malaria vaccine targets. The three major phases of the *P*. *falciparum* life cycle that can be broken using vaccines to aid in eliminating malaria include pre-erythrocytic, blood-stage, and transmission-blocking phases [25]

Even though numerous new drug compounds have been found to treat malaria, drug resistance development raises concerns about the effectiveness of the currently available medications [18, 38]. Creating a potential malaria vaccine is crucial to addressing such issues and is anticipated to effectively eradicate malaria worldwide [30]. A vaccine that functions with maximum efficacy and efficiency, acting in a manner that first inhibits the pathogen’s earliest growth phase before its subsequent stages, is the most efficient strategy to stop the spread of malaria. Figure 2 [25] shows the three major phases of the *P*. *falciparum* life cycle that can be broken using vaccines to prevent transmission and eliminate malaria. The three phases include pre-erythrocytic, blood-stage, and transmission-blocking phases [25]. Making individuals immune to infectious diseases is one of the essential aspects of international health measures, especially in comparison to other human activities like cleanliness and sanitation [10]. According to a recent phase IIb clinical trial with the identifier NCT03896724, the antigen R-21, a malaria vaccine candidate developed using Matrix-M adjuvant from Novavax Inc. by the University of Oxford, showed 77% effectiveness in children [9].

However, the research on implementing the first malarial vaccine ordered by the World Health Organization (WHO) is the most noteworthy undertaking that has been completed in this respect [10]. The most progressive malaria vaccine is the RTS, S vaccine [30], with only around 36% efficacy. The modest efficacy of this existing Mosquirix vaccine curtails malaria eradication efforts. The results from Mosquirix clinical trials demonstrated that after 4 years of follow-up, the vaccine’s effectiveness against malaria was modest, just 36% among kids aged 5 to 17 months who received four doses [25]. Similarly, Arora, Anbalagan, and Pannu [3] point out that RTS, S/AS01 is ineffective against types of malaria caused by *P. vivax*, *P. knowlesi*, *P. ovale*, and *P. malariae*. Mosquirix vaccine’s modest efficacy and ineffectiveness against certain types of malaria provide a scientific gap. Vaccines with better efficacies should be developed, or adjuvants that can increase the effectiveness of the existing Mosquirix vaccine should be discovered to aid in fighting malaria. This study seeks to explore and fill this scientific gap by using in-silico approaches to find compounds from natural sources (ZINC database) that can increase the efficacy of the existing Mosquirix vaccine.

It is acknowledged that vaccines can induce defense from novel infections by inducing robust cellular and humoral immune reactions against *Plasmodium*’s sporozoite phase in the mammal host. However, the fundamental mechanisms provoking immune reactions to malaria pre-erythrocytic phases are not entirely comprehended. While the vaccinated host produces sporozoite-specific antibodies that prevent sporozoite invasion of the hepatocytes [28], satisfactory amounts of sporozoite antigen-specific T lymphocytes aid in the elimination of the liver cells infected with the *Plasmodium* parasites that display these epitopes [23]. The associations between the host hepatocytes and sporozoite depend on the circumsporozoite protein (CSP), a crucial component of the sporozoites’ surface coat [30]. As a result, the CSP can potentially be a target antigen in anti-malarial vaccines for the pre-erythrocytic stage. This study used CSP as a target molecule to discover compounds that can enhance the RTS, S vaccine’s efficacy and its public health benefits.

Circumsporozoite surface protein (CSP) is the most prevalent polypeptide in the sporozoite covering. This protein functions in the sporozoite’s motility and invasion as it enters the hepatocyte [42]. Fernández-Arias et al. [12] outline that a team led by Victor Nussenzweig and Ruth S. discovered circumsporozoite as a significant surface protein of *P. berghei* sporozoites. Following this, CSPs of additional plasmodial species were found and demonstrated to have comparable structural and immunological characteristics. An immunodominant B cell epitope’s random repeat surrounded by C- and N-terminal domains make up the CSP, which has an estimated size of 40–60 kDa [12].

In the salivary invasion and maturation process in the vector and human liver cells, the CSP of the infective sporozoite of all *Plasmodium* species can be evidenced. The finding of sequence variation in the repeated sequence of its core part gene forced a re-evaluation of this strategy. However, it has been a prominent target in creating recombinant malaria vaccines [42]. The central repeat region (CRR) and the conserved domains RI (positioned in the amino-terminal) and RII (situated in the carboxyl-terminal) are present in all CSPs [42]. Being an essential component of *P*. *falciparum* sporozoites, CSP has been used to create a malaria vaccine (Mosquirix) that generates monoclonal antibodies (mAbs) against CSP. L9 is one of the mAbs that target PfCSP or can be considered anti-PfCSP. Discovering other molecules that target PfCSP can help eradicate malaria by improving the efficacy of the Mosquirix vaccine.

**Fig. 3:**
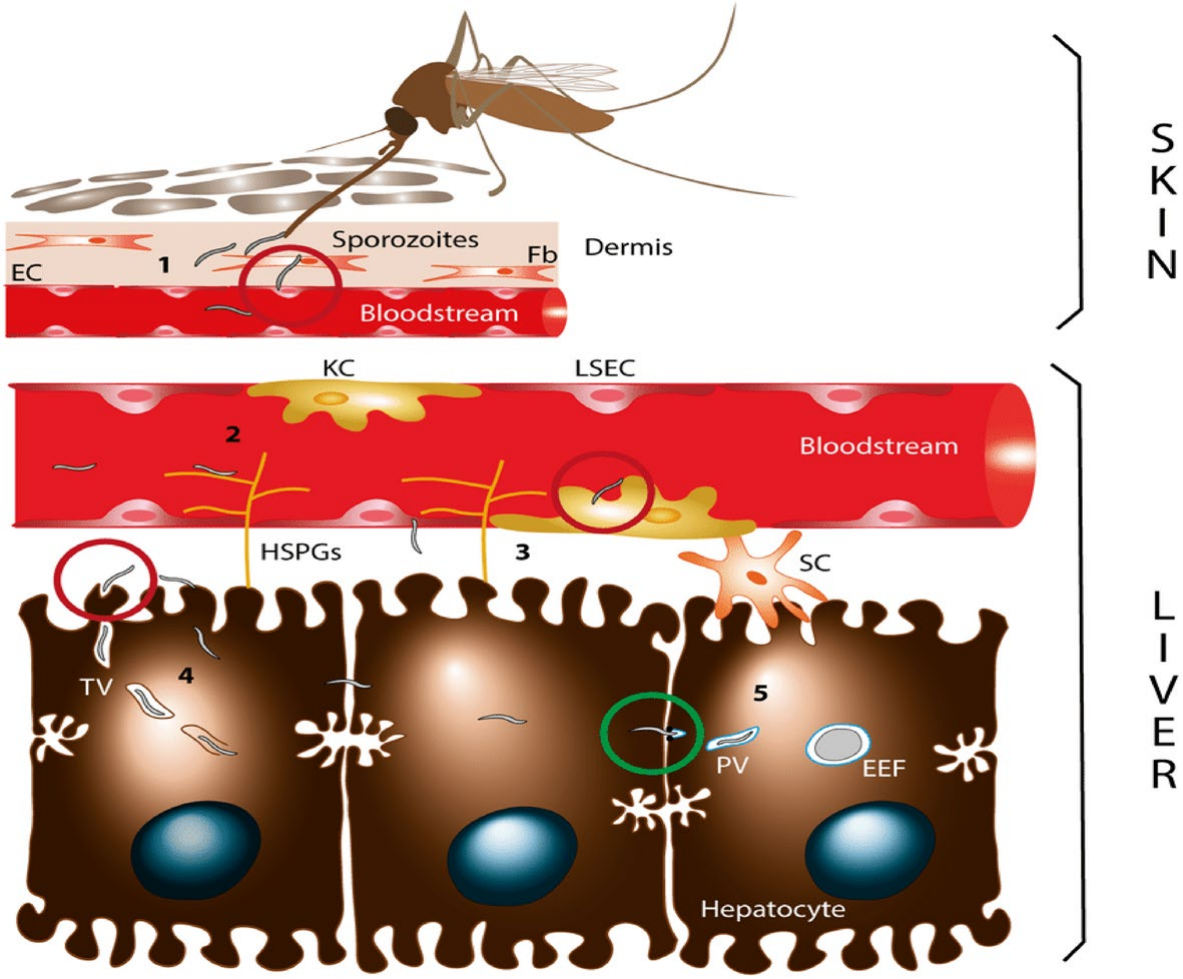
CSP functional cycle. (1) An infected mosquito deposits sporozoites in the mammalian host dermis via a bite. (2) The sporozoites enter the bloodstream by crossing the dermal fibroblasts (Fb) and endothelial cells (EC). They then traffic to the liver. The sporozoites are sequestered in the liver sinusoids through the association between hepatic heparan sulfate proteoglycans (HSPGs) and CSP. (3) Those sporozoites, through Kupffer cells (KCs) and liver sinusoidal endothelial cells (LSECs), cross the sinusoidal cellular barrier into the liver parenchyma. (4) The sporozoites traverse through numerous liver cells, sometimes inside a transient vacuole (TV). (5) A parasitophorous vacuole (PV) formation, in which the parasite matures into a replicative exoerythrocytic form (EEF), enables the sporozoites to switch to the productive invasion of a final hepatocyte [27]

In human trials, it was discovered that the RTS, S/AS01 vaccine produced cellular and humoral responses that protected mosquito bite challenge infections [28]. Along with protecting against malaria, the vaccine also produced defensive immune reactions against hepatitis B [48]. Different researchers have developed some monoclonal antibodies against CSPs of other plasmodial species using Mosquirix. Most of them have been demonstrated to identify CSP immunodominant repeat domain and to counteract parasite infection in vitro and, in some instances, *in vivo* [12]. Notably, one study used a phage display library to successfully recover a monoclonal antibody against CSP from a person exposed to *P*. *falciparum* sporozoites [12]. This study sought to explore the possibility of discovering other effective compounds that can increase the efficacy of the Mosquirix vaccine by characterizing novel PfCSP inhibitors with pharmacological properties on Mosquirix through hierarchical virtual screening using monoclonal antibody L9 as the ligand/reference structure. Figure 3 [27] displays the functional cycle of CSP in malaria development, providing avenues that can be exploited to discover anti-CSP compounds.

Several scholars have also identified PfCSP as a malaria drug and vaccine target. Therefore, it is not surprising that the RTS, S/AS01 recombinant, CSP-based vaccine is one of the most well-known anti-malarial subunit vaccines. A lesser amount of aspartic acid and valine residues can be found in the 58 kDa CSP protein, which also has 37–49 NANP (N, asparagine; A, alanine; P, proline) amino acid repeats [28]. Marques-da-Silva et al. [28] outline that multiple human leukocyte antigens DR isotype (HLA-DR) compounds can detect the T cell epitopes of the CSP C-terminal region. The RTS, S/AS01 vaccine comprises immunodominant CD8 and CD4 T cell epitopes, a B-cell receptor epitope, NANP amino acid repeat sequence, and the NF54 strain of *P. falciparum* CSP central repeat region merged to hepatitis B surface antigens [28]. To aid the immunogen’s ability to self-assemble into virus-like particles, three times as much “free” HBsAg antigen (S) is added to the vaccine preparation. By causing significant levels of CD4 T cells that express the co-stimulatory marker CD40L, IFN-, TNF, and IL-2 and anti-CSP antibodies, RTS, S/AS01 vaccines protect against malaria [28].

**Fig. 4:**
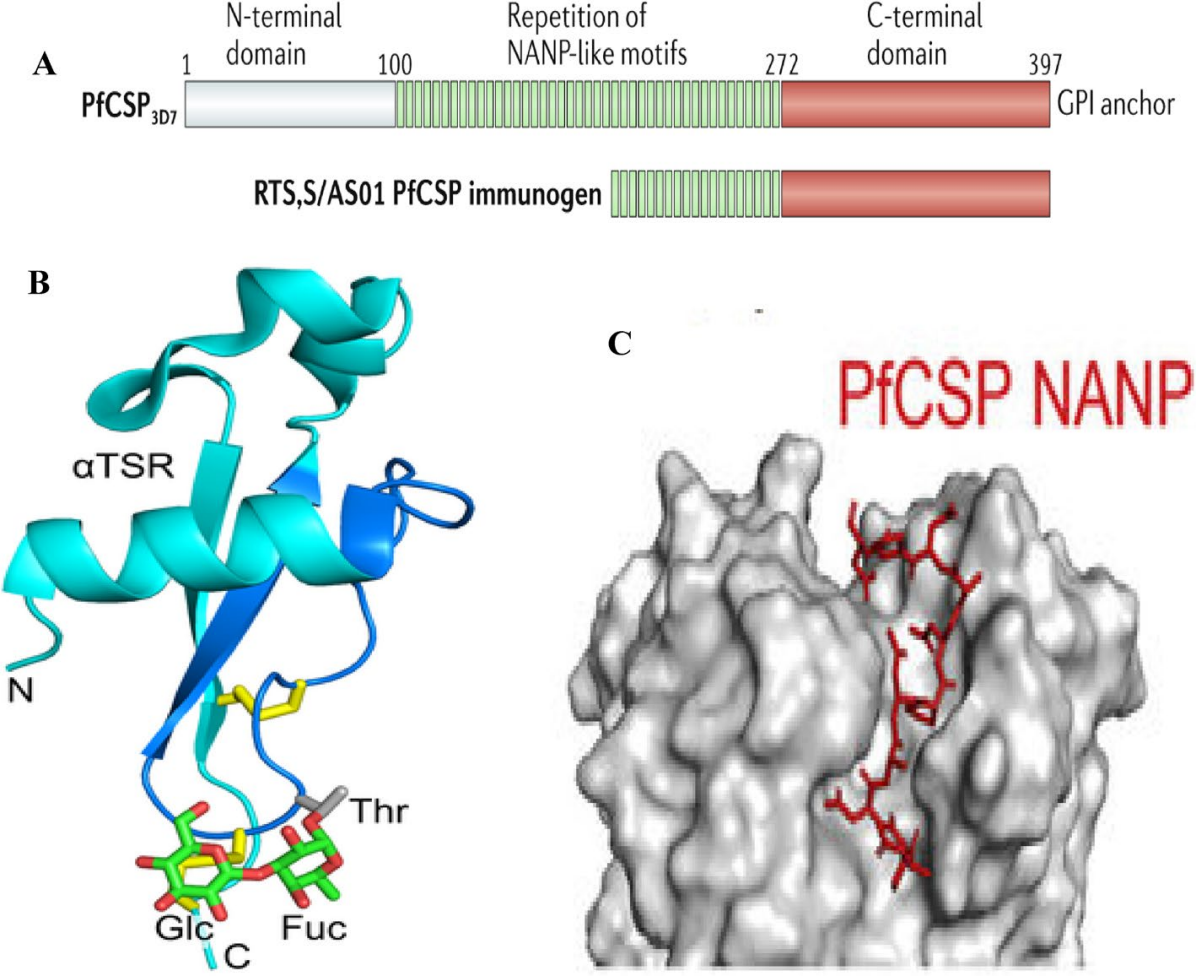
PfCSP domain organization and structure view. **A** Schematic model of the different PfCSP domains labeled as N-terminal domain, NANP repetition domain, and C-terminal domain. The figure also shows the Mosquirix PfCSP immunogen that shares structural similarities to PfCSP [20]. **B** Mass spectrometry observed a 3D model of the TSR domain of CSP displaying glycosylation location. Glycans are represented using sticks with green denoting carbon and red signifying oxygens. Silver carbons and blue nitrogens represent amino acid side chains attached to glycans. Yellow denotes disulfide bonds [43]. **C** Surface representation of how antibodies interact with PfCSP by binding to its NANP repeats to prevent sporozoites from invading hepatocytes [10]

Since PfCSP is necessary for the central surface protein on infectious sporozoites (SPZ) to infect hepatocytes, it is the ideal vaccine target. As shown in Fig. 4 [10], a C-terminus, a repeated tetrapeptide core domain, and an N-terminus are the three domains that makeup PfCSP [51]. The region at the intersection of the repeat domain and N-terminus in the Pf3D7 reference strain begins with NPDP and is followed by three repeats that alternate between NANP and NVDP. Thirty-five NANP repeats follow this “junctional zone,” with a fourth NVDP added after the 20th NANP [8]. According to structural analyses, the three tetrapeptides joined to form DPNA, NPNV, and NPNA are the motifs that PfCSP mAbs recognize in the repetition domain [34]. Notably, RTS, S only has the C-terminus and 19 NANP repeats [51]. All counteracting PfCSP mAbs described in existing literature target the immunodominant NANP repeats [20].

Identifying uncommon and powerful mAbs that bind NPDP [22, 45] or the NVDP repeats [50] highlighted these subdominant epitopes as weak spots on PfCSP. These discoveries resulted in the creation of next-generation vaccines against the junctional area because these epitopes are not seen in RTS, S [4, 5, 15, 19]. Scholars have lately identified which epitopes can enhance immunogen design and choose the most effective mAb for medical progress by examining the binding and efficacy of a panel of defensive human PfCSP mAbs. The panel’s most effective protective mAb was mAb L9, which primarily binds NVDP repeats and cross-reacts with NANP repeats. Isothermal titration calorimetry (ITC) revealed that L9 and other powerful mAbs bound recombinant PfCSP (rPfCSP) in two binding occurrences with different attractions, raising the possibility that this *in vitro* hallmark of “two-step binding” might be associated with *in vivo* sporozoites neutralization [50]. Therefore, PfCSP was used as the target protein to characterize novel PfCSP inhibitors with pharmacological properties on Mosquirix through hierarchical virtual screening using monoclonal antibody L9 as the ligand/reference structure.

**Fig. 5:**
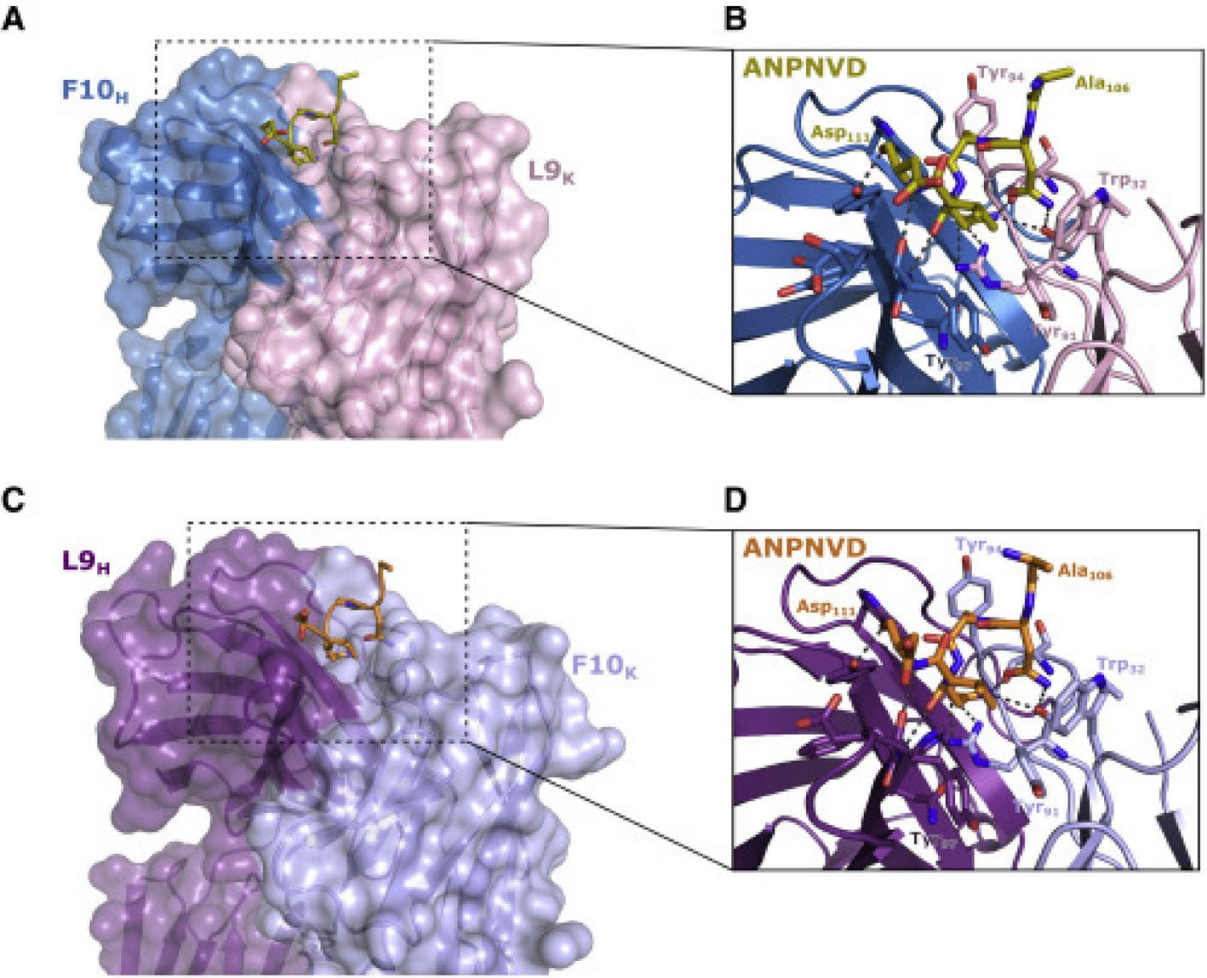
L9 as a PfCSP inhibitor. **A** and **C** Transparent surface representation with a visible cartoon representation of F10HL9k Fab (**A**) structures and L9_H_F10_k_ Fab (**C**) bound to NANPNVDP. **B** and **D** Zoomed-in images of the binding sites in F10_H_L9_k_ Fab (**B**) and L9_H_F10_k_ Fab (**D**). The stick representations show the peptide interacting residues, with the dashed lines signifying hydrogen bonds [51]

L9 is a powerful human monoclonal antibody (mAb) that binds to PfCSP on malaria-infective sporozoites and cross-reacts with significant NANP repeats. It is easier to develop vaccines if the ontogeny and PfCSP binding mechanisms of this mAb are understood. Wang et al. [51] isolated mAbs with a clonal affinity for L9 and demonstrated how this B-cell lineage initially exhibits NVDP affinity before developing NANP reactivity. Combining the L9 kappa light chain (L9) with clonally associated heavy chains creates chimeric mAbs that cross-link two NVDP, react with NANP, and kill sporozoites *in vivo* more effectively than their light chain-only counterparts [51]. The chimeric mAbs bound minor repeats in a type-1 turn similar to other repeat-specific antibodies, according to structural studies undertaken by Wang et al. [51]. These findings demonstrate the critical role L9 plays in binding NVDP to PfCSP to kill sporozoites and imply that PfCSP-based immunogens may benefit from the presentation of 2 NVDP [51]. In this regard, mAb L9 was essential in the study as a reference ligand for virtual screening to characterize novel PfCSP inhibitors with pharmacological properties on Mosquirix. Figure 5 shows monoclonal antibody L9 as a PfCSP inhibitor and its interaction with PfCSP.

Traditional vaccine and drug design and development methods have been employed for several years during drug discovery. However, these conventional methods of developing drugs and vaccines are expensive and time-consuming [32]. Therefore, there is a paradigm shift from these traditional drug and vaccine development techniques to the rapidly emerging in-silico approaches that are revolutionizing vaccine and drug discovery. Some in-silico tools for developing vaccines include ANTIGENpro, AllergenFP, AllerTOP, and others [39]. Researchers have used such in-silico vaccine design tools and techniques to develop vaccine candidates. For instance, Khalid et al. [21] created a rational vaccine design utilizing the method of epitope mapping to curb the infections caused by *A. baumannii*. The authors combined epitopes of an outer membrane protein with immunogenic potential (target protein) to form a 240-amino-acid vaccine sequence that underwent different processes to act as a vaccine candidate [21]. On the same note, Rodrigues-da-Silva et al. [36] used in-silico methods to validate *P. vivax* malaria vaccine candidate merozoite surface protein-9 (MSP9) and support its inclusion in future subunit vaccines. This study is in tandem with those reported by Takashima et al. [44], all of which use in-silico processes to narrow down the candidate list of novel transmission-blocking vaccines discovered directly using human malaria parasites. Using in-silico approaches to designing vaccines can lead to an effective fight against infections and diseases.

During the pre-clinical phase of vaccine and drug research and development, it is crucial to carry out additional *in vitro* and *in vivo* validation of the therapeutic potential of the potential drugs and vaccine candidates after discovering them. For instance, to determine the true potential of doxycycline (DOX) against COVID-19, Sachdeva et al. [37] suggested more *in vitro* and *in vivo* investigations. Depending on the pathogen and disease of interest, *in vitro* drug sensitivity testing is frequently employed. The many drug sensitivity assays used for antimalarial medication efficacy testing that target distinct phases of the parasite’s growth were highlighted in a systematic review conducted by Sinha et al. in 2017. According to Sinha et al. [40], some of these tests include HTS like fluorescence-based assay and the gametocyte stage tests such as SMFA. Other tests include blood stage tests like radioisotopic assay.

The SYBR green assay is one of the most popular antimalarial drug sensitivity assays. Because of its dependability as a drug screening and monitoring tool, it is regarded as the GOLD standard for *in vitro* malaria drug sensitivity testing [7]. The 50% inhibitory concentrations (IC_50_) of clinical isolates have been calculated using a straightforward and affordable technology, according to Cheruiyot et al. [7]. This assay has been utilized in studies by researchers, such as Traoré et al. [46], which evaluated the susceptibility of *P. falciparum* isolates to antimalarial medications in Mali. Similar to this, Duan et al. [11] used the SYBR green test to assess the susceptibilities of *P. falciparum* isolates to 11 antimalarial medications. Therefore, the effectiveness of the chosen PfCSP inhibitors was assessed using this test.

## Methods

### PfCSP structure retrieval and preparation

Using the PDB ID 3VDL, the 3D structure of PfCSP was located and downloaded in PDB format from the Protein Data Bank (PDB) database (https://www.rcsb.org). The structure was prepared for molecular docking using BIOVIA Discovery Studio 2021 by removing all side chains, water molecules, heteroatoms, and bound ligands, leaving behind only chain A. Polar hydrogens were added. PfCSP 3D structure was generated and saved as a .pdb file.

### Retrieval and preparation of monoclonal antibody L9 structure

L9's 3D structure was obtained from the Protein Data Bank (PDB) using PDB ID 7RQP and downloaded in PDB format. It was loaded into BIOVIA Discovery Studio 2021, and all side chains and ligands were eliminated, leaving only the chain B (L9 Light Kappa Chain). The L9 antibody’s retrieved 3D structure was stored as a .pdb file.

### Pharmacophore-based virtual screening

The L9 antibody was utilized to identify active compounds with comparable structures that can inhibit PfCSP in the ZINCPHARMER web server (http://zincpharmer.csb.pitt.edu/pharmer.html). These active compounds were put through drug-likeness tests and ADMET property analysis to see if they may be employed as antimalarial adjuvants.

### Drug-likeness test and ADMET property analysis

The drug-likeness test and pharmacokinetics analysis were carried out using the SwissADME web application (http://www.swissadme.ch/). The active compounds’ SMILES were copied and pasted into the SwissADME web server. Muegge, Egan, Veber, Ghose, and Lipinski's Rule were among the drug-likeness filters used. Similarly, bioavailability radars and the Brain Or IntestinaL EstimateD permeation (BOILED-Egg) diagram were used to examine pharmacokinetic results. For molecular docking, active compounds that met all of the bioavailability and permeability requirements, as well as at least four drug-likeness filters, were chosen.

### Molecular docking

The PfCSP inhibitors were docked with PfCSP using Autodock Vina, a built-in tool in the PyRx program. Using the PyRx program, PfCSP was converted from a .pdb to .pdbqt format. The chosen ligands were prepared for molecular docking by minimizing their energies and converting them to .pdbqt format with the PyRx program. All protein-ligand complexes with the lowest binding energies following molecular docking were chosen as the final potential therapeutic candidates.

### Molecular dynamics simulation (MDS)

GROMACS 2022 was employed. The topology files for the ligand and protein as well as parameter files for the ligand were created using the Charmm 36 Force Field in CHARMM-GUI web server (https://www.charmm-gui.org/). The default settings of the CHARMM-GUI web server were preferred, including water box size options, the number of ions to be added to the protein-ligand complexes, ion addition method (Monte-Carlo ion putting method), and system temperature (300.00K). The number of steps in the GROMACS energy minimization method was set to 5000. A 100 ps run was used to equilibrate the minimized system. The final production run was set at 100 ns. GROMACS was used to calculate the number of hydrogen bonds, root mean square fluctuation (RMSF), and root mean square deviation (RMSD) after the last run.

### In vitro validation of PfCSP inhibitors

The potential Mosquirix adjuvants were tested *in vitro* using the SYBR green assay to determine their inhibitory capabilities and antimalaria activity. The candidate adjuvants were diluted to a concentration of 200 ng/ml in 300 μl of dimethyl sulfoxide (DMSO). Using *Plasmodium* parasite growth culture, whose preparation is described in in vitro Module WWARN procedure INV02 [17], this starting concentration was serially diluted to 11 dilutions in a 96-well plate (12 columns by 8 rows); 150 μl of each dilution was pipetted into a fresh plate. Each drug-coated plate well got 150 μl of a 1% parasitemic sample. After 3 days of culture in a closed environment at 37^o^C, the parasite-loaded plate was removed from the incubator and 150 μl of lysis solution containing a DNA intercalating dye (SYBR green 1) was added to the wells. At room temperature, the plate was incubated. The Tecan machine was then used to measure the fluorescence/absorption of surviving parasites. The inhibitory concentration 50 (IC_50_) was calculated using the wavelength measurements from this test. For analysis, Microsoft Excel was utilized, which converted the data read from the Tecan machine into a graph. The generated R2 value was significant. A R2 value close to one indicates that the regression line is a perfect match for the data and may be used to determine IC_50_ values.

## Results

### PfCSP structure retrieval and preparation

**Fig. 6:**
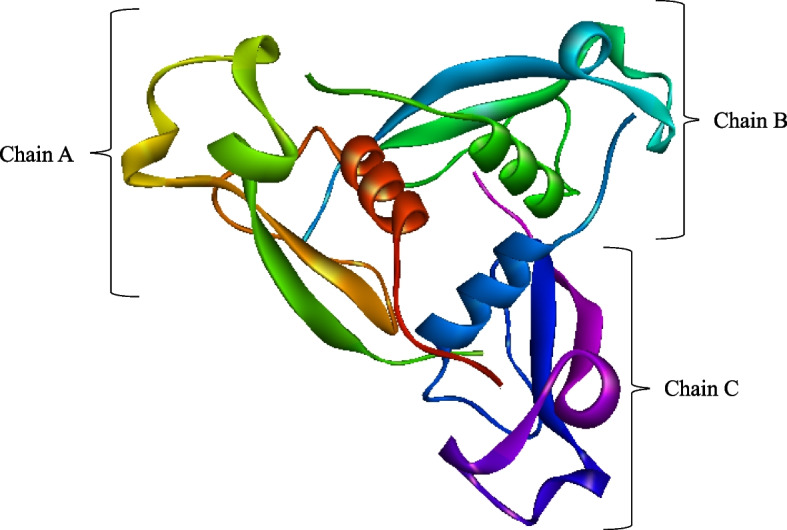
The 3D structure of prepared PfCSP. PfCSP retrieved from PDB, ID 3VDL. All heteroatoms and water molecules removed and polar hydrogens added. The three chains, A, B, and C, are indicated

PDB ID 3VDL was used to retrieve the 3D structure of PfCSP from the PDB database and prepared for molecular docking in BIOVIA Discovery Studio 2021 by removing all bound compounds except chain A (Fig. 6). Polar hydrogens were added to chain A of PfCSP. The three chains, A, B, and C, of PfCSP are shown in Fig. 6.

### Retrieval and preparation of monoclonal antibody L9 structure

**Fig. 7:**
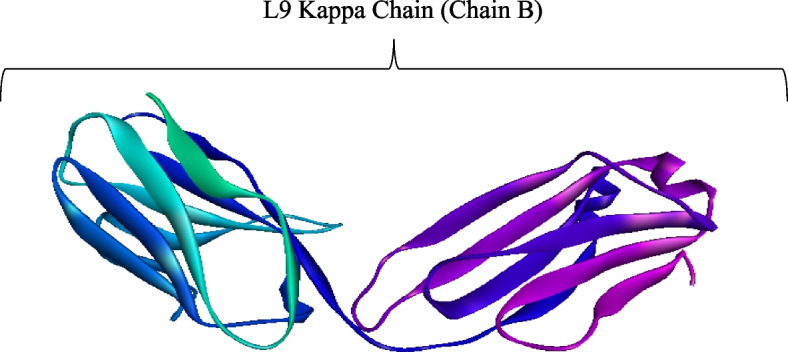
The 3D structure of prepared L9 Kappa chain. It was retrieved from PDB, ID 7RQP. All side chains and ligands removed

The 3D structure of L9 antibody was retrieved from PDB database using PDB ID 7RQP. During preprocessing in BIOVIA Discovery Studio 2021, it was discovered that L9 antibody has two chains, A (L9 Heavy Chain) and B (L9 Light Kappa Chain). Chain A was deleted from the 3D structure, leaving behind only chain B that is responsible for PfCSP binding, shown in Fig. 7.

### Pharmacophore-based virtual screening

mAb L9 was loaded into the ZINCPHARMER web server. Six L9’s features, namely one aromatic ring (interacting with THR 172), one hydrogen donor (interacting with SER 12), two hydrogen acceptors (interacting with LEU 11 and SER 12), and two hydrophobic amino acids (interacting with THR 172 and THR 10) were selected to create a pharmacophore. The pharmacophore was used to perform virtual screening, yielding 23 hits. Table 1 shows the 23 hits obtained from the pharmacophore-based virtual screening process. The 23 hits were downloaded and saved in a .sdf file.

**Table 1:** Basic information on the virtual screening results

No.	Molecule	RMSD	Mass	RBnds
1	ZINC04529323	0.286	493	8
2	ZINC32780968	0.625	493	7
3	ZINC32780968	0.625	493	7
4	ZINC22939614	0.642	534	9
5	ZINC40144754	0.441	395	5
6	ZINC13152865	0.362	485	8
7	ZINC40144754	0.440	395	5
8	ZINC39933536	0.666	472	8
9	ZINC07948710	0.434	377	5
10	ZINC67103919	0.658	370	8
11	ZINC25374360	0.582	449	8
12	ZINC25374360	0.574	449	8
13	ZINC22938754	0.643	492	8
14	ZINC32735690	0.434	396	7
15	ZINC71998971	0.288	433	6
16	ZINC57991640	0.571	448	9
17	ZINC67410702	0.678	374	8
18	ZINC67410702	0.675	374	8
19	ZINC70705715	0.651	618	12
20	ZINC12530088	0.494	472	8
21	ZINC17588493	0.383	446	7
22	ZINC71996727	0.427	420	4
23	ZINC09125912	0.487	460	9

### Drug-likeness test and ADMET properties analysis

**Fig. 8:**
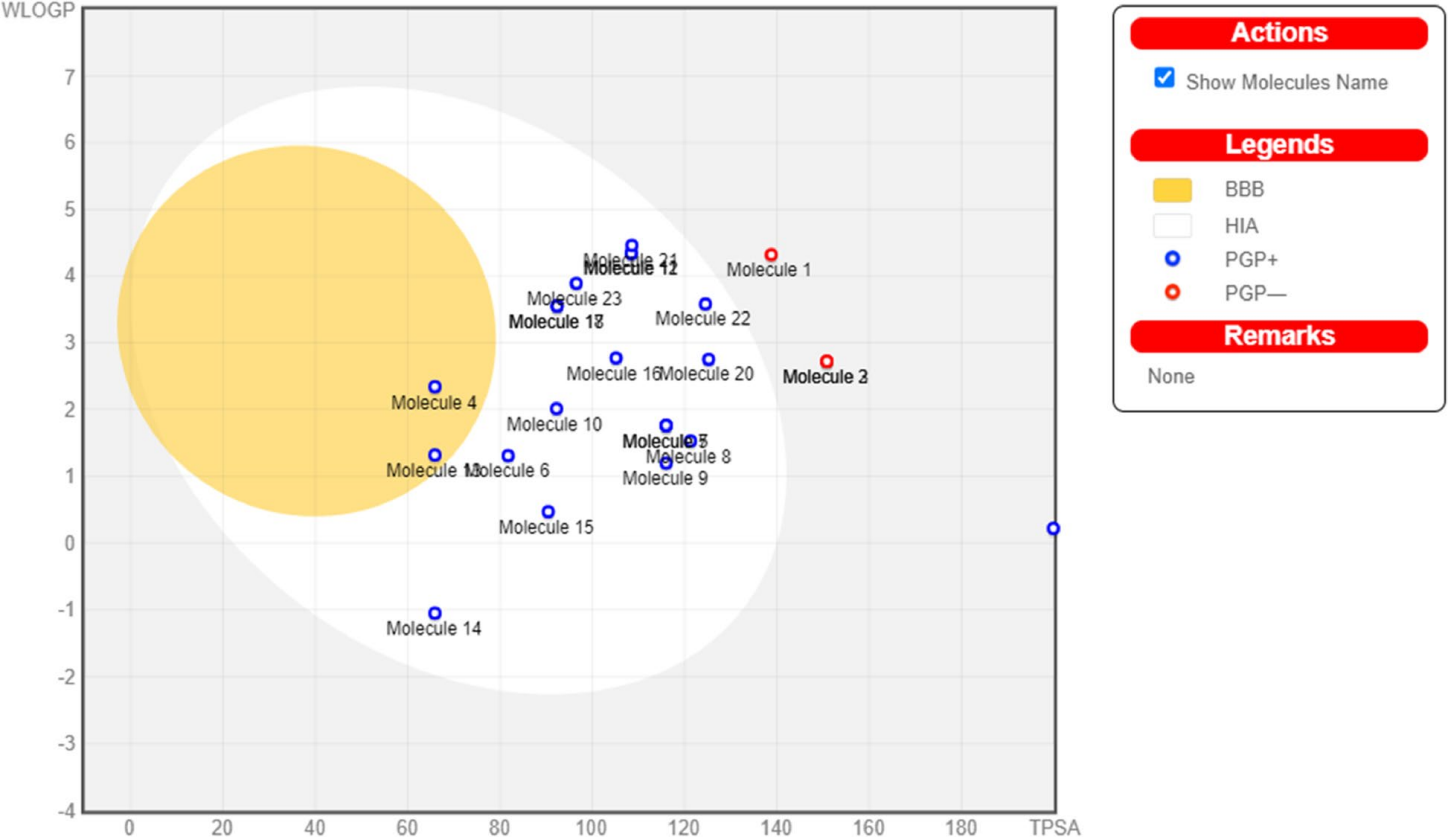
BOILED-Egg Analysis (L9 Antibody). Boiled egg prediction of blood–brain barrier permeability and gastrointestinal absorption for the 23 hits. Four molecules (1, 2, 3, and 19) are out of range, thus excluded. The other molecules are P-glycoprotein (P-gp) substrate, indicated by the blue dot, depicting their ease of excretion from the body

**Fig. 9:**
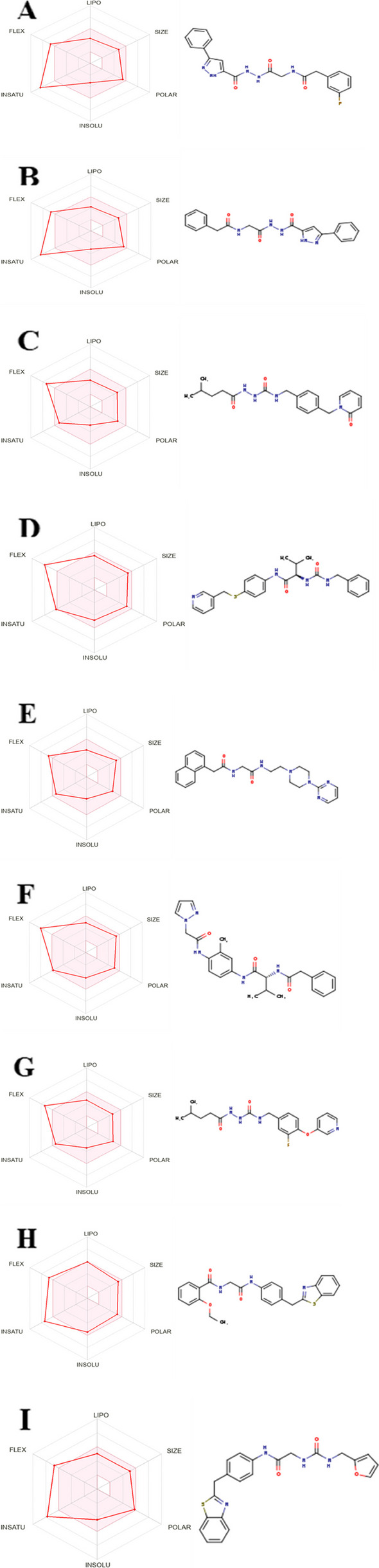
The structures and oral bioavailability radars of the 9 hits (L9 antibody). **A **ZINC40144754, **B** ZINC07948710, **C** ZINC67103919, **D** ZINC25374360, **E** ZINC71998971, **F** ZINC57991640, **G** ZINC67410702, **H** ZINC17588493, and **I** ZINC71996727

The virtual screening hits were subjected to a drug-likeness test to determine drug-ability and a pharmacokinetics analysis to determine oral bioavailability. Table 2 shows that 12 of the 23 hits satisfied at least four of the five drug-likeness filters. From the 12 compounds with drug-likeness properties in this study, it was discovered that three were duplicates and removed: ZINC40144754, ZINC25374360, and ZINC67410702. When subjected to pharmacokinetics analysis, ZINC04529323, ZINC32780968, ZINC32780968, and ZINC70705715 were out of the required range, as evident in the BOILED-Egg analysis chart (Fig. 8). These four molecules had already been excluded from further analysis because they did not satisfy the drug-likeness requirements. Ultimately, only nine hits had drug-likeness characteristics and good pharmacokinetics properties in this study (Fig. 9).

**Table 2:** Drug-likeness test results of the 23 molecules

No.	Molecule	Lipinski	Ghose	Veber	Egan	Muegge	Drug-like?
1	ZINC04529323	Yes	No	Yes	No	Yes	No
2	ZINC32780968	Yes	No	No	No	No	No
3	ZINC32780968	Yes	No	No	No	No	No
4	ZINC22939614	Yes	No	No	Yes	No	No
5	ZINC40144754	Yes	Yes	Yes	Yes	Yes	Yes
6	ZINC13152865	Yes	No	No	Yes	Yes	No
7	ZINC40144754	Yes	Yes	Yes	Yes	Yes	Yes
8	ZINC39933536	Yes	No	No	Yes	Yes	No
9	ZINC07948710	Yes	Yes	Yes	Yes	Yes	Yes
10	ZINC67103919	Yes	Yes	No	Yes	Yes	Yes
11	ZINC25374360	Yes	Yes	No	Yes	Yes	Yes
12	ZINC25374360	Yes	Yes	No	Yes	Yes	Yes
13	ZINC22938754	Yes	No	No	Yes	Yes	No
14	ZINC32735690	Yes	No	No	Yes	Yes	No
15	ZINC71998971	Yes	No	Yes	Yes	Yes	Yes
16	ZINC57991640	Yes	Yes	No	Yes	Yes	Yes
17	ZINC67410702	Yes	Yes	No	Yes	Yes	Yes
18	ZINC67410702	Yes	Yes	No	Yes	Yes	Yes
19	ZINC70705715	No	No	No	No	No	No
20	ZINC12530088	Yes	No	No	Yes	Yes	No
21	ZINC17588493	Yes	Yes	Yes	Yes	Yes	Yes
22	ZINC71996727	Yes	Yes	Yes	Yes	Yes	Yes
23	ZINC09125912	Yes	No	No	Yes	Yes	No

### Molecular docking

**Fig. 10:**
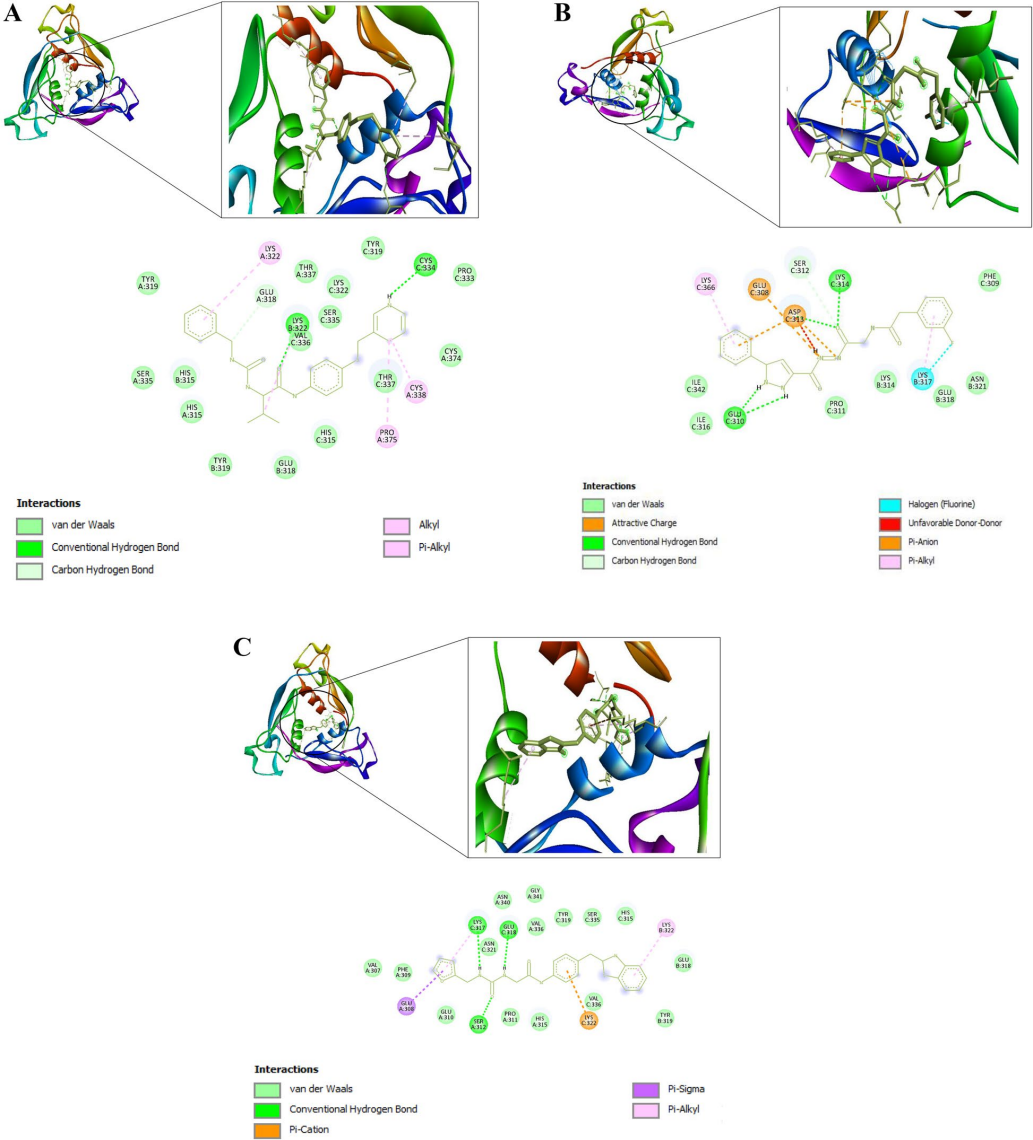
3D and 2D interactions of PfCSP and the 3 ligands. **A** PfCSP and ZINC25374360, with binding affinity of −8.1 kcal/mol. **B** PfCSP and ZINC40144754, with binding affinity of −8.3 kcal/mol. **C** PfCSP and ZINC71996727, with binding affinity of −8.9 kcal/mol

The prepared PfCSP (PDB ID 3VDL) was loaded into the PYRX software as a .pdb file and converted into a .pdbqt molecule for docking purposes. A file containing the 9 lead compounds in the .sdf format was then loaded into the PYRX software. The energy of all the 9 ligands was minimized. The ligands were then converted into the preferred .pdbqt format. Docking was then done and the results were as follows: ZINC40144754 (−8.3), ZINC07948710 (−7.7), ZINC67103919 (−7.5), ZINC25374360 (−8.1), ZINC71998971 (−8.0), ZINC57991640 (−7.7), ZINC67410702 (−6.9), (H) ZINC17588493 (−7.8), and ZINC71996727 (−8.9). No docking conformation was found when docking mAb L9 to PfCSP. Three lead compounds were chosen for molecular dynamics simulation based on their binding affinities, which was below −8.0. They include ZINC40144754 (−8.3), ZINC25374360 (−8.1), and ZINC71996727 (−8.9). Figure 10 shows the interaction between the three candidate Mosquirix adjuvants with PfCSP assessed using BIOVIA Discovery Studio 2021. From the assessment of PfCSP-ZINC40144754 interaction, it was discovered that the candidate Mosquirix adjuvant does not interact with chain A. Further analysis discovered that ZINC40144754 forms unfavorable donor–donor interactions with PfCSP (Fig. 10). Therefore, it was excluded from the molecular dynamics simulation process because of these unfavorable characteristics that might affect its stability when in complex with PfCSP. Only ZINC25374360 and ZINC71996727 were the final candidate Mosquirix adjuvants and were subjected to molecular dynamics simulation.

### Molecular dynamics simulation (MDS)

**Fig. 11:**
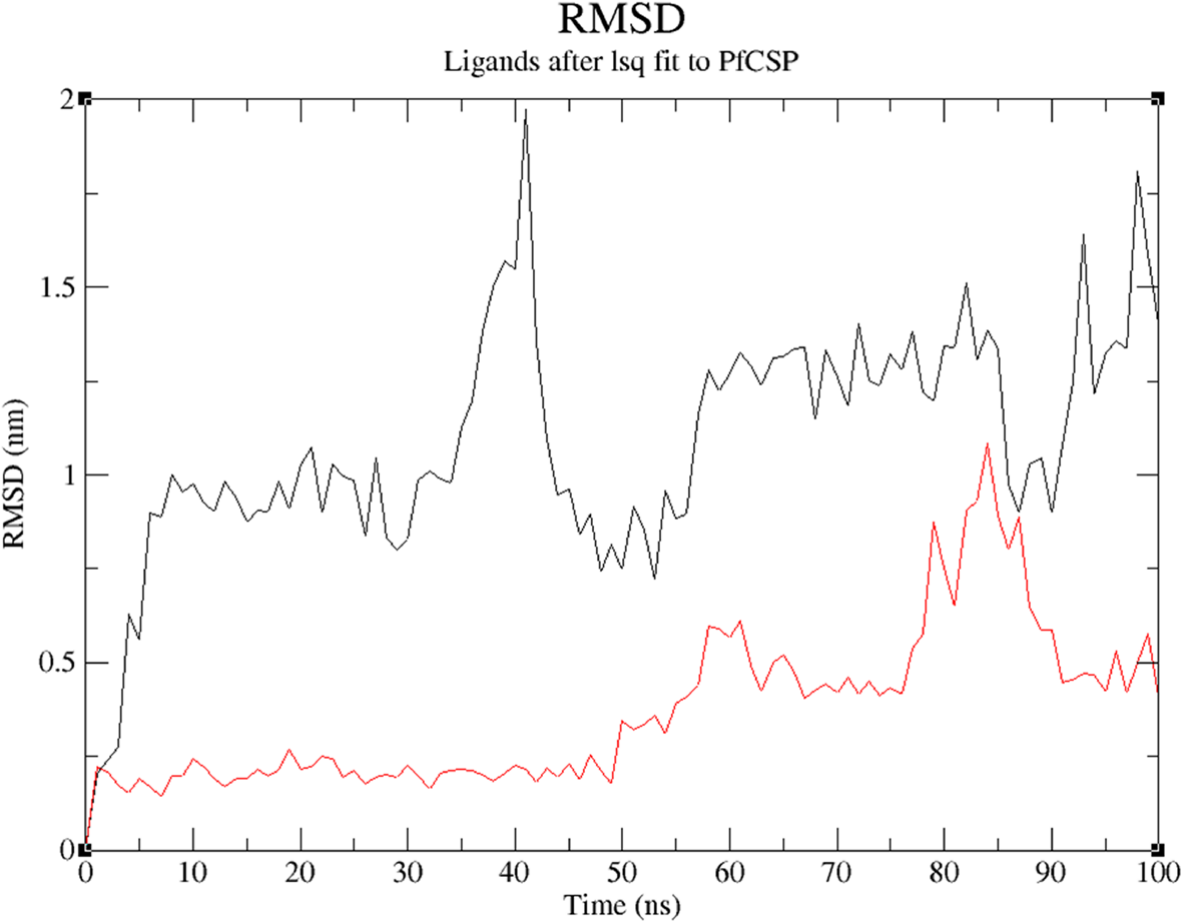
RMSD plot of PfCSP (PDB ID: 3VDL) with the two ZINC database compounds as a function of 100 ns simulation time. ZINC25374360 (black) and ZINC71996727 (red)

**Fig. 12:**
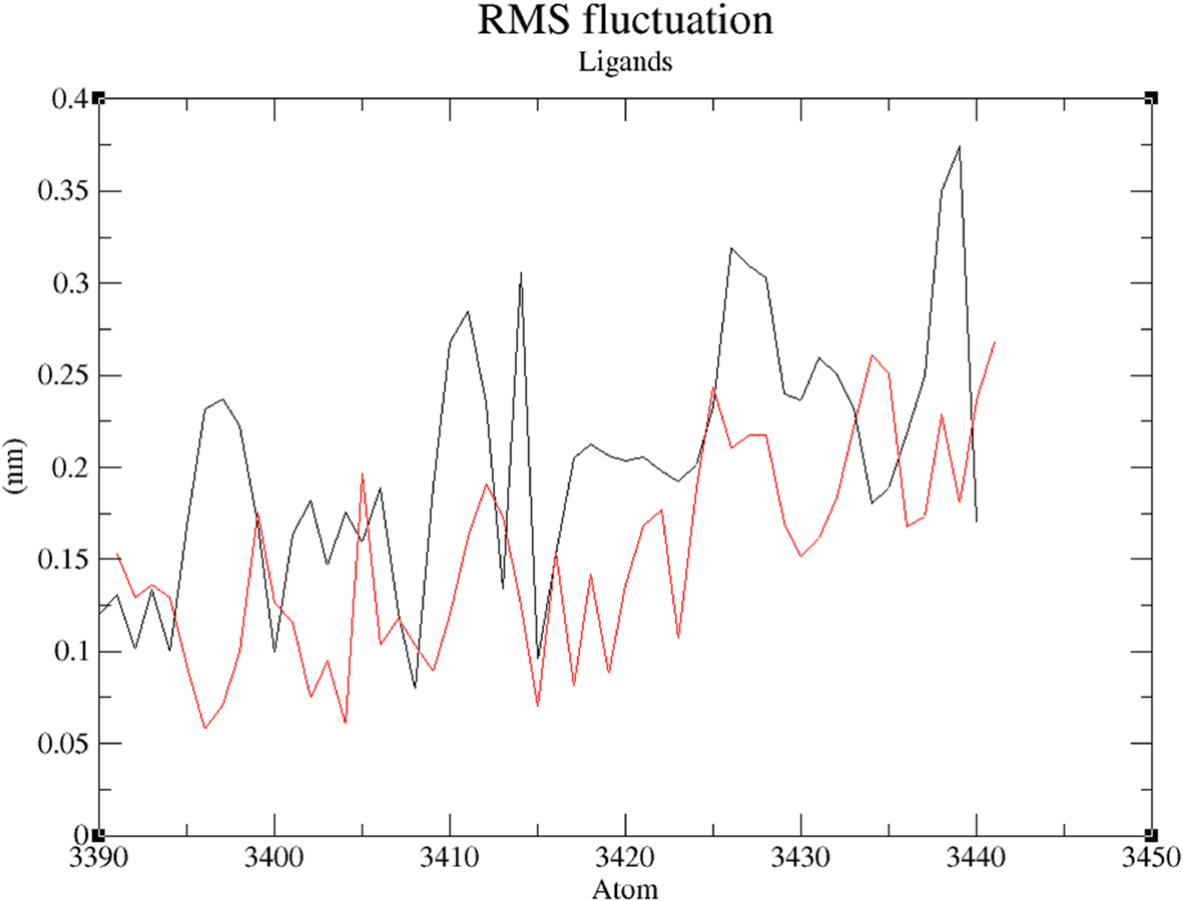
RMSF plot of the two ZINC database compounds. ZINC25374360 (black) and ZINC71996727 (red)

**Fig. 13:**
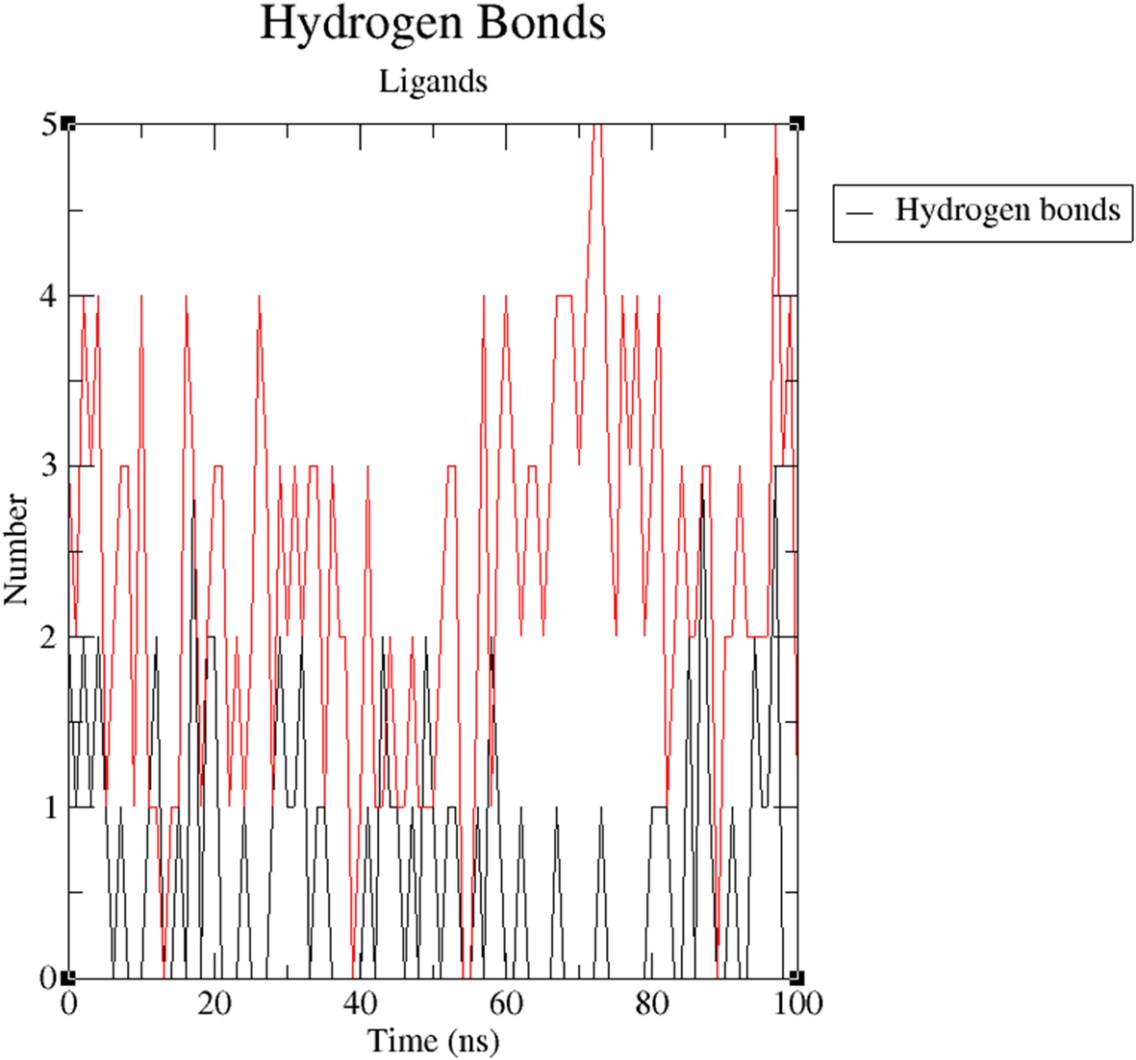
Number of hydrogen bonds plot. The number of hydrogen bonds between PfCSP (PDB ID: 3VDL) with the two ZINC database compounds as a function of 100 ns simulation time. ZINC25374360 (black) and ZINC71996727 (red)

Figure 11 shows how ZINC25374360 and ZINC71996727 did not experience major deviations. For the first 10 ns, ZINC25374360 experienced a deviation of approximately 1 nm. It then stabilized for around 25 ns before deviating with an approximate distance of 1 nm within a 10-ns period. From 45 to 100 ns, ZINC25374360 remained relatively stable with minor deviations of an average distance of 0.25 nm. ZINC71996727 is more stable than ZINC25374360 because of its negligible deviations. It gains stability within the first 50 ns. Even though it deviates a bit for the last 50 ns, the deviations are negligible with an average distance of approximately 0.5 nm. The atoms of the two ZINC compounds fluctuate within an acceptable distance of 0.2 nm (Fig. 12). Throughout the 100-ns simulation, ZINC25374360 forms between 0 and 3 hydrogen bonds with PfCSP while ZINC71996727 forms approximately 1 to 5 hydrogen bonds with the same target protein (Fig. 13). These RMSF, RMSD, and hydrogen bond results prove that ZINC25374360 and ZINC71996727 form stable conformations with PfCSP.

### In vitro validation of candidate Mosquirix adjuvants

**Fig. 14:**
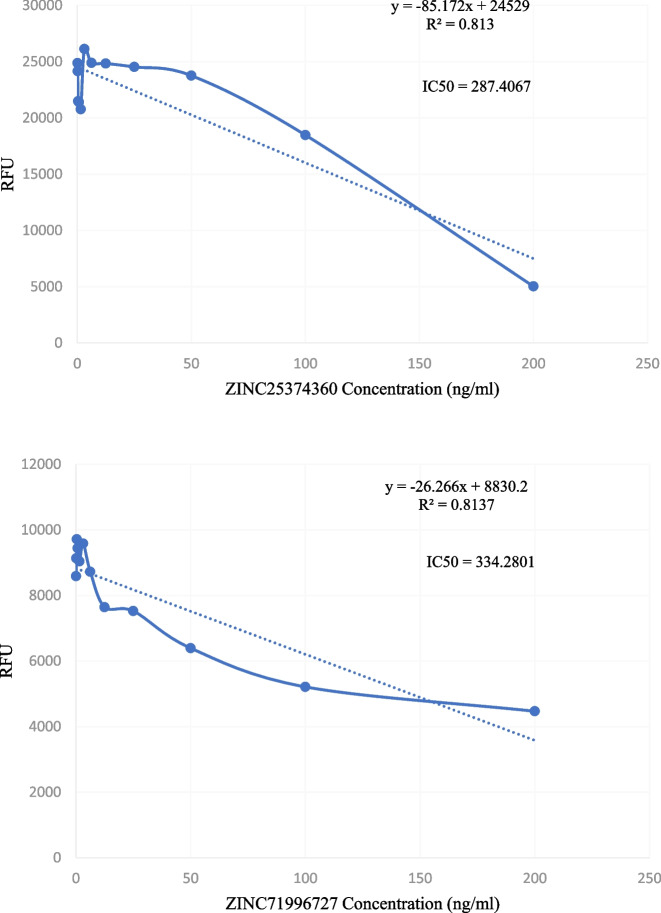
Point to point plot. IC50 value determination of PfCSP inhibitors using point-to-point calculation

Following MDS, the inhibition ability of the two candidate Mosquirix adjuvants was assessed through *in vitro* experiment using the SYBR green assay. Point-to-point calculation of the IC_50_ values of the two candidate adjuvants showed that at concentration between 250 and 350 ng/ml, ZINC25374360 (287.41 ng/ml) and ZINC71996727 (334.28 ng/ml) (Fig. 14), they can inhibit the growth of *P*. *falciparum*.

## Discussion

With the existence of malaria vaccine, Mosquirix, a substantial decrease in the rates of malaria mortality and morbidity should be evident. However, malaria is still causing significant fatality because of the modest efficacy of Mosquirix, creating a need for novel malaria vaccines with better efficacies. While the application of traditional vaccine development strategies is still rampant, in silico approaches to vaccine design and development is gaining popularity. For instance, epitope mapping is commonly being used to design vaccines [21]. Therefore, this study used in silico approaches, hierarchical virtual screening and molecular dynamics simulation, to mine potent malaria vaccine adjuvant leads that can increase the efficacy of Mosquirix from the ZINC database.

### PfCSP structure retrieval and preparation

PfCSP, the target protein, was retrieved from PDB database using PDB ID 3VDL (Fig. 6) and pre-processed utilizing BOIVIA Discovery Studio 2021 by removing all bound ligands. These compounds were eliminated because they did not contribute in the binding of PfCSP to the potential Mosquirix adjuvants. Deleting them resulted in a more ideal posture search and simplified computations that would have been difficult if such compounds had muddled PfCSP’s binding pockets. Polar hydrogens were added to chain A of PfCSP to help locate hydrogen bond interactions in the 3D structure. Hydrogen bond interactions are critical for determining the binding affinity of the potential Mosquirix adjuvants to PfCSP.

Target protein preparation is a crucial step in drug design and discovery as evident in studies performed by Alzain et al. [1] and Gao et al. [16]. While identifying novel *Plasmodium falciparum* dihydroorotate dehydrogenase (PfDHODH) inhibitors for malaria, Alzain et al. [1] downloaded the crystal structure of the protein using PDB ID 7KZ4 and prepared it for docking by creating disulfide bonds, adding hydrogens, removing water beyond 5 Å from hetero groups, creating zero-order metal bonds, and creating het states with Epik to ensure PfDHODH had the ideal structural orientation for docking.

Similarly, to discover small-molecule inhibitors targeting SARS-CoV-2 main protease (M^pro^), Gao et al. [16] retrieved the M^pro^ protein from PDB using ID 7KX5 and preprocessed it for molecular docking in the protein preparation wizard, Schrödinger Suite 2022-2, by deleting non-water solvents, water molecules beyond 8 Å of the binding ligand, and co-crystalized ions and metals. They deleted and re-added the protein’s hydrogens and reassigned the bond orders to achieve the protein’s conformational integrity suitable for molecular docking [16]. Therefore, preprocessing of PfCSP done in this study is in line with what other studies have done.

### Retrieval and preparation of monoclonal antibody L9 structure

Figure 7 shows the 3D structure of chain B of L9 antibody retrieved from PDB database. It was utilized as a ligand in the virtual screening of natural compounds database, ZINC database, to find compounds with similar structural orientation and potential anti-malarial characteristics or activities. Wang et al. [51] outline that the L9 light chain (chain B) is crucial for binding the minor repeats in PfCSP and preventing malaria.

As evident in other studies, it is uncommon to use such a large protein like mAb L9, which is not commercially available in small ions or molecules databases like PubChem, as a ligand in a drug design and discovery process. Most ligands are often commercially available small ions, molecules, compounds, or chemicals. When performing a ligand-based virtual screening to find new human papillomavirus 16 type inhibitors, Razzaghi-Asl et al. [35] used jaceosidin, a small compound available in the PubChem database, as the ligand. Similarly, Oduselu et al. [31] used pyridoxal 5′-phosphate as a ligand to perform pharmacophore-based virtual screening to identify *Plasmodium falciparum* 5-aminolevulinate synthase inhibitors.

Even though the properties of the ligand used in this study is not in line with the ones reported in other studies, mAb L9 was preferred because it is the most effective proactive mAb produced following administration of Mosquirix vaccine, and it primarily binds NVDP repeats and cross-reacts with NANP repeats in PfCSP [50]. Its affinity to the minor repeats in PfCSP makes it a suitable molecule to be used in virtual screening for the discovery of potential Mosquirix adjuvants.

### Pharmacophore-based virtual screening

Since mAb L9 is a very large protein, it was discovered that it has several stereochemical features, which could limit the number of hits obtained during virtual screening, when its 3D structure was loaded into the ZINCPHARMER web server. Some of its features were used to develop a pharmacophore that was used to perform virtual screening yielding 23 hits (Table 1). This is consistent with several studies that created a pharmacophore model from either one large compound or several ligands before performing pharmacophore-based virtual screening.

Oduselu et al. [31] used pyridoxal 5′-phosphate to construct an effective pharmacophore query for virtual screening based on four key ligands’ properties: aromaticity, hydrophobicity, hydrogen bond donors, and hydrogen bond acceptors. The authors obtained 2755 hits from the pharmacophore-based virtual screening. Instead of using one ligand, Onyango et al. [32] created a pharmacophore model using eight ligands. The subsequent pharmacophore-based virtual screening process yielded 18,009,471 hits [32].

### Drug-likeness test and ADMET properties analysis

The drug-likeness test determines the suitability of a compound to be used as a drug. The 12 candidate Mosquirix adjuvants in Table 2 satisfied at least four of the five drug-likeness filters in SwissADME web server. These drug-likeness filters aid in the identification of molecules with favorable medicinal characteristics. Drug-likeness test is an essential step in drug design and discovery through in-silico means [33]. Even though this study used five drug-likeness filters, others use only one as in the case of Razzaghi-Asl et al. [35] who utilized only the Lipinski’s rule of five to create a drug-likeness profile of the candidate ligands obtained during virtual screening; 2246 out of 2819 compounds had suitable drug-likeness properties in that study [35].

ADMET property analysis is frequently performed concurrently with drug-likeness test to determine the pharmacokinetics properties of candidate drugs. The BOILED-Egg analysis chart (Fig. 8) shows molecules with good oral bioavailability and permeation in small blue circles while molecules with inappropriate pharmacokinetics properties are displayed in small red circles. ADMET property analysis estimates pharmacokinetics properties and toxicity risk of potential drugs [33]. Razzaghi-Asl et al. [35], Onyango et al. [32], and Oduselu et al. [31] performed ADMET properties analysis to determine the absorption, distribution, metabolism, excretion, and toxicity profiles of their drug candidates. Figure 9 shows the nine hits with appropriate drug-likeness characteristics and good pharmacokinetics properties in this study.

### Molecular docking

The molecular docking process was initiated to determine the binding affinities of the nine hits to PfCSP. The complex formed between PfCSP and mAb L9 was to be used as the reference. However, the docking process proved that no structural conformation exists between PfCSP and mAb L9, which might be the cause of its modest efficacy. Possible factors for the unsuccessful docking between mAb L9 and PfCSP might be low binding affinity of the complex [13, 49], essential bound-like conformations being away from the complementarity-determining region (CDR) loop conformations, and L9 antibody undergoing large conformational changes between its bound and unbound experimental structure [13, 14]. Therefore, candidate Mosquirix adjuvants with binding energies lower than −8.0 kcal/mol were selected for MDS. At that binding energy, it is believed that the candidate Mosquirix adjuvant leads bind strongly to PfCSP. While that might be true, further visualization of the interaction between the candidate Mosquirix adjuvants is necessary. In this current study, the visualization showed unfavorable binding between one of the final three candidate adjuvants (ZINC40144754) to PfCSP (Fig. 10B). Therefore, it was excluded from subsequent MDS.

### Molecular dynamics simulation (MDS)

MDS was used to corroborate the docking results and examine the behavior of the final candidate Mosquirix adjuvants within PfCSP’s binding pocket. The two candidate Mosquirix adjuvants deviate at an acceptable average distance of 0.25 nm (Fig. 11). Therefore, both compounds form complexes with PfCSP that have low risk of conformational changes. Even though ZINC25374360 and ZINC71996727 did not have a reference ligand used for comparison purposes, the movement of their atoms could be assessed based on acceptable fluctuation distances. The results give an acceptable average RMSF value of 0.2 nm (Fig. 12), proving that the two candidate Mosquirix adjuvants do not undergo high divergence from their average positions. Oduselu et al.’s [31] and Razzaghi-Asl et al.’s [35] studies ensured RMSF values of their protein-ligand complexes are within acceptable levels to infer their stability. This shows the importance of ZINC25374360 and ZINC71996727 maintaining a low RMSF value during the simulation in this study. Hydrogen bond analysis further confirms the stability of the two complexes. Throughout the 100 ns simulation, ZINC25374360 forms three hydrogen bonds while ZINC71996727 forms five hydrogen bonds with PfCSP (Fig. 13). The hydrogen bond analyses demonstrate that the candidate Mosquirix adjuvants maintain stable conformation in PfCSP’s active site during the 100 ns simulation, suggesting their inhibitory potential. Similarly, these number of hydrogen bonds confirm that the two ZINC database compounds bind strongly to PfCSP.

### In vitro validation of candidate Mosquirix adjuvants

Following MDS, *in vitro* validation has become a common strategy in contemporary drug design and discovery processes [33]. Since several assays exist for drug sensitivity testing, various researchers use different assays in their studies. Cheng et al. [6] used ADP-Glo kinase assay to determine the capability of specific compounds in inhibiting p38γ activity. In this study, the SYBR green assay was utilized to assess the inhibitory potential of the two candidate Mosquirix adjuvants. Using chloroquine as a benchmark antimalarial compound, these two ZINC compounds emerge as Mosquirix adjuvant leads that can increase the efficacy of the malaria vaccine. Chloroquine is selected as a suitable benchmark antimalarial compound because of its minimal toxicity, long duration of action, quick onset, and high tolerance in humans [53]. It also treats susceptible infections with a broad spectrum of *Plasmodium* species, including *P*. *malariae*, *P*. *vivax*, *P*. *ovale*, and *P*. *falciparum* [53].

The average IC_50_ for chloroquine assay with 1% parasitemia against the 3D strain of *P. falciparum*, according to Molnár et al. [29], is 34.68 ± 5.28 nM. Using the formula (nM) = (ng/mL)/(MW in KD), this IC_50_ value equates to 17.89 ng/ml. A quick comparison between IC_50_ of chloroquine with those of the two candidate Mosquirix adjuvants (Fig. 14) suggests that the two ZINC compounds are needed in higher concentrations to treat malaria than chloroquine. Even though this is not ideal, at those very high concentrations, the two PfCSP inhibitors may still increase the efficacy of Mosquirix vaccine by enhancing the binding affinity of mAb L9 to PfCSP and prevent malaria. However, additional homogenizing assessment has to be done to ensure the adjuvants and the Mosquirix vaccine can exist uniformly as one antimalarial substance, structural optimization studies to determine whether the adjuvants can prolong the binding of mAb L9 to PfCSP, and clinical testing using *in vivo* techniques to ascertain the efficacy of the PfCSP inhibitors as Mosquirix adjuvants in humans.

## Conclusions

Mosquirix vaccine has failed to prevent and eradicate malaria because of its modest efficacy. Similarly, its inability to act effectively against other *Plasmodium* species other than *P*. *falciparum* makes it an unsuitable malaria prevention strategy. Therefore, ways of increasing its efficacy and effectiveness and broadening the scope of its action are urgent. With existing in silico approaches to drug and vaccine design and discovery, these needs can be satisfied. This study performed hierarchical virtual screening through both pharmacophore-based approach and molecular docking to discover potent candidate Mosquirix vaccine adjuvants. Further MDS and *in vitro* studies were undertaken to ascertain the inhibitory potential of the virtual screening hits obtained. Eventually, ZINC25374360 and ZINC71996727 emerged as the most potent Mosquirix vaccine adjuvants. They can be used to increase the efficacy of the Mosquirix vaccine. However, further homogenization analysis, *in vivo* tests, and structural optimization studies are necessary to determine whether these two candidates Mosquirix adjuvants can increase the efficacy of the malaria vaccine and provide health benefits to humans.

## Data Availability

The datasets used and/or analyzed during the current study are available from the corresponding author on reasonable request.

## Abbreviations

PfCSP: *Plasmodium falciparum* circumsporozoite surface protein
WHO: World Health Organization
mAb: Monoclonal antibody
RMSD: Root means square deviation
MDS: Molecular dynamics simulation
CSP: Circumsporozoite surface protein
kDa: Kilodalton
CRR: Central repeat region
DNA: Deoxyribonucleic acid
HLA-DR: Human leukocyte antigens DR isotype
CD4: Cluster of differentiation 4
CD8: Cluster of differentiation 8
NANP: Asparagine, alanine, asparagine, proline
DPNA: Aspartic acid, proline, asparagine, alanine
NPNV: Asparagine, proline, asparagine, valine
NPNA: Asparagine, proline, asparagine, alanine
NPDP: Asparagine, proline, aspartic acid, proline
NVDP: Asparagine, valine, aspartic acid, proline
CD40L: Cluster of differentiation 40 ligand
IFN: Interferons
IL-2: Interleukin 2
Fb: Fibroblasts
EC: Endothelial cells
HSPG: Hepatic heparan sulfate proteoglycans
KCs: Kupffer cells
LSECs: Liver sinusoidal endothelial cells
TV: Transient vacuole
PV: Parasitophorous vacuole
EEF: Exoerythrocytic form
SPZ: Sporozoites
ITC: Isothermal titration calorimetry
rPfCSP: Recombinant PfCSP
IC_50_: Half maximal inhibitory concentration
DOX: Doxycycline
MSP9: Merozoite surface protein-9
PDB: Protein database
